# Supplementary material for: Rapid processing of neutral and angry expressions within ongoing facial stimulus streams: Is it all about isolated facial features?
Source: PLoS One. 2020 Apr 24;15(4):e0231982. doi: 10.1371/journal.pone.0231982 (PMC7182236; doi:10.1371/journal.pone.0231982)
Supplement: S1 File — (DOCX) [file pone.0231982.s001.docx]

Supplementary materials

# Emotion ratings

At the end of each experiment, we asked participants to judge the level of perceived emotional valence of the faces, i.e., how intensely they thought each face conveyed anger, happiness, or disgust. Stimuli were presented for the duration of one cycle at the stimulation frequency, i.e., 67 msec after *Pilot 1* and *2* and 167 msec after *Experiment 1* and *2*. Participants used a Self-Assessment-Manikin [[1]](https://www.zotero.org/google-docs/?LNyqNs) ranging from 1 (very low emotional intensity) to 9 (very high emotional intensity). Specific questions were administered to verify that participants interpreted each emotional expression not only as being different from neutral, but also conveying the intended emotion [[2]](https://www.zotero.org/google-docs/?ZHMziM). An overview of the ratings for each experiment, question, and condition can be found in *S1 Table* (see also *S1 Fig*).

We carried out Bayesian multilevel ordinal regressions using *brms* [[3]](https://www.zotero.org/google-docs/?6LNAf8). The ordinal variable (i.e., ratings on a 9-point Likert scale) was analyzed with models including emotional expression as constant effect, whereas intercepts and slopes were allowed to vary according to participant and picture. As likelihood function, a cumulative model with *probit* link (i.e., assuming the latent variable to be normally distributed) was applied [[4]](https://www.zotero.org/google-docs/?PtQBlO). All other settings were identical to the analysis of EEG data. Separate models were fitted for each experiment and question. Differences between conditions were assessed by computing the mean and the 95% highest density interval (HDI) of the difference between posterior distributions of the parameters of interest [[5]](https://www.zotero.org/google-docs/?LDHqAg) and calculating evidence ratios (ERs).

Results unambiguously showed that, in all experiments, angry faces were perceived as conveying more anger than neutral and happy faces. Similarly, happy faces were rated as happier than neutral and angry faces. Finally, disgusted faces were considered as conveying more disgust than neutral, angry, and happy faces (see *S2 Table*). Thus, the emotional expressions in the selected facial stimuli were perceived by our participants in accordance with normative ratings.

# Reaction Times

In all experiments except *Experiment 2*, participants were instructed to press the spacebar on a standard QWERTZ USB keyboard any time they detected, within the area of the face, a target turquoise dot (RGB: 128, 128, 196; diameter = 0.3° of visual angle). See *S3 Table* for an overview of the reaction times recorded for each experiment and condition. As a working exploratory hypothesis, we surmised that reaction times ought to be slower when targets overlapped with regularly presented emotional faces, because predictable emotional information would automatically capture attention away from the task. We also hypothesized an interaction with stimulus orientation, given that emotional features should be more difficult to extract from faces presented upside down. It would also be plausible to assume that high within-identity emotion variability would make it more difficult to efficiently process emotional cues in such a short presentation time.

We discarded reaction times of incorrect responses and imputed missing averages using the *R* package *mice* [[6]](https://www.zotero.org/google-docs/?OTJEVh). For the analyses, we fitted Bayesian multilevel models including, as constant effect, the combinations of face orientation (upright, inverted) and regularity (neutral, angry, irregular) condition levels. We additionally allowed intercepts to vary as a function of participant, to model interindividual differences related to response speed. We also allowed slopes to vary per participant, to model the possibility that behavioral performance would be differentially modulated by face orientation and regularity. All models were fitted using weakly informative priors (in msec), i.e., *Normal(500,300)* on beta coefficients and *HalfCauchy(50)* [[7,8]](https://www.zotero.org/google-docs/?VjEjQ9) on the standard deviation of varying effects. All other settings were identical to the analysis of EEG data.

## Results

### Pilot 1

Reaction times in upright and inverted conditions were similar, with only a slight increase for irregular inverted (as opposed to upright) faces [~11 msec, ER = 11.61]. Reaction times were also comparable in all regular and irregular conditions (see *S4 Table*).

### Pilot 2

Upright and inverted faces elicited comparable reaction times. The only exception was slower reaction times for inverted faces in the irregular condition, only when within-identity emotion variability was low [~72 msec, ER = 132.20].

Reaction times during trials with inverted, low variability faces were faster for regular neutral faces as compared to regular angry [~33 msec, ER = 18.06] and irregular [~68 msec, ER = 94.29].

Finally, for upright faces responses were slower in high compared to low within-identity emotion variability in regular neutral [~42 msec, ER = 50.31] and irregular trials [~57 msec, ER = 14.88].

### Experiment 1

Upright and inverted faces elicited comparable reaction times, except for slower responses in trials with inverted regular neutral [~40 msec, ER = 10.31] and inverted irregular faces [~56 msec, ER = 25.64] when within-identity emotion variability was low.

Trials with irregular faces resulted in slower reaction times compared to regular angry [~58 msec, ER = 65.70] and neutral faces [~50 msec, ER = 30.76] when emotion variability was high and faces presented upright. In trials with inverted faces and low variability, only trials with angry regular faces showed faster responses than irregular trials [~50 msec, ER = 22.82]. In addition, this condition also resulted in faster reaction times relative to neutral [~63 msec, ER = 82.38].

Finally, when comparing high vs. low within-identity emotion variability, upright irregular conditions elicited slower reaction times [~80 msec, ER = 332.50] for high emotion variability. When faces were inverted, angry upright faces with high emotion variability resulted in slower responses [~38 msec, ER = 11.81].

## Discussion

In *Pilot 1*, reaction times were comparable in all regular and irregular conditions. In *Pilot 2*, upright and inverted faces elicited similar reaction times, except with inverted faces when no emotion regularity was shown and within-identity emotion variability was low.

In *Experiment 1*, inverted stimuli elicited slower responses in trials with regular neutral and irregular faces as compared to upright faces, but only when within-identity emotion variability was low. Trials with no regularity generally resulted in slower reaction times compared to regular conditions, particularly when emotion variability was high. Emotion regularity also resulted in faster responses compared to irregular conditions, especially in situations of high emotion variability. When comparing high vs. low within-identity emotion variability, upright irregular conditions elicited slower reaction times, whereas responses to angry upright faces were slower when presented upside-down.

Thus, some of these findings could tentatively be interpreted as suggesting that emotional cue extraction is more difficult for inverted stimuli, no emotion regularity, and high within-identity emotion variability, thereby leading to behavioral disadvantages in an unrelated detection task. However, given the exploratory nature of the analyses, the low number of participants (especially in the pilot studies), and the noisy estimates (i.e., very broad 95% HDI), we advise caution in the interpretation. In the future, interested researchers may wish to systematically evaluate the reliability of this behavioral measure in this and other tasks during RSVP streams.

# Stimulation frequency

The strength of intertrial phase clustering (quantified by cosine similarity, *CS*) should not be different across conditions, given that no regularity was presented at the respective stimulation frequencies (i.e., 15 Hz for *Pilot 1* and *2*, 6 Hz for *Experiment 1* and *2*). Regarding the influence of orientation and within-identity emotion variability, we had no *a priori* hypothesis. Statistical analyses were identical to the ones conducted for the regularity frequencies (see main text for details).

## Results

### Pilot 1

Face inversion or emotion regularity did not reliably modulate CS (see *S6 Table*).

### Pilot 2

CS was not modulated by face inversion or regularity. However, faces with high (vs. low) within-identity emotion variability resulted in larger CS for all conditions, except inverted irregular [ER = 3.70].

### Experiment 1

Face inversion modulated CS. Specifically, when within-identity emotion variability was high, regular angry and irregular trials elicited larger CS when upright [ER = 44.45 and 201.53, respectively]. When variability was low, only irregular trials showed larger CS when upright [ER = 34.16].

Regular neutral and angry as well as irregular conditions elicited similar CS in all orientation and variability conditions. The only exception was irregular compared to neutral trials when orientation was upright and emotion variability was high [ER = 37.65].

Finally, high within-identity emotion variability elicited larger CS than low variability in all conditions.

### Experiment 2

When within-identity emotion variability was high, all regular and irregular trials elicited larger CS when upright compared to inverted. When variability was low, only irregular trials showed larger CS when upright [ER = 14.66].

When directly comparing the different regularity conditions separately for all orientation and variability conditions, regular neutral, regular angry, and irregular conditions elicited similar CS.

High within-identity emotion variability elicited larger CS than low variability only in upright regular angry [ER = 84.56] and irregular conditions [ER = 10.88].

## Discussion

While CS in *Pilot 1* was not modulated by condition, *Pilot 2* revealed larger values for high relative to low within-identity emotion variability in all regular conditions. In *Experiment 1*, emotion variability also interacted with face orientation, with regular angry and irregular trials eliciting larger CS when upright and emotion variability was high. *Experiment 2* showed a similar pattern of results, with larger CS when faces were upright and variability was high. In particular, upright regular angry and irregular conditions elicited larger CS when variability was high.

Taken together, these results might tentatively hint at increased attention allocation during highly variable (upright) face streams, especially for regular angry and irregular conditions. However, just as mentioned for reaction times, we prefer to exercise caution with the interpretation of these findings, given their heterogeneous pattern.

# References

[1. Bradley MM, Lang PJ. Measuring Emotion: The Self-Assessment Mannequin and the Semantic Differential. J Behav Ther Exp Psychiatry. 1994;25: 49–59.](https://www.zotero.org/google-docs/?sQAWUV)

[2. Tottenham N, Tanaka JW, Leon AC, McCarry T, Nurse M, Hare TA, et al. The NimStim set of facial expressions: Judgments from untrained research participants. Psychiatry Res. 2009;168: 242–249. doi:10.1016/j.psychres.2008.05.006](https://www.zotero.org/google-docs/?sQAWUV)

[3. Bürkner P-C. brms: An R Package for Bayesian Multilevel Models Using Stan. J Stat Softw. 2017;80: 1–28. doi:10.18637/jss.v080.i01](https://www.zotero.org/google-docs/?sQAWUV)

[4. Bürkner P-C, Vuorre M. Ordinal Regression Models in Psychology: A Tutorial. Adv Methods Pract Psychol Sci. 2019; 2515245918823199. doi:10.1177/2515245918823199](https://www.zotero.org/google-docs/?sQAWUV)

[5. Kruschke JK. Doing Bayesian Data Analysis: A Tutorial with R, JAGS, and Stan. 2nd edition. Boston: Academic Press; 2014.](https://www.zotero.org/google-docs/?sQAWUV)

[6. van Buuren S, Groothuis-Oudshoorn K. mice: Multivariate Imputation by Chained Equations in R. J Stat Softw. 2011;45: 1–67. doi:10.18637/jss.v045.i03](https://www.zotero.org/google-docs/?sQAWUV)

[7. Gelman A. Prior distributions for variance parameters in hierarchical models (comment on article by Browne and Draper). Bayesian Anal. 2006;1: 515–534. doi:10.1214/06-BA117A](https://www.zotero.org/google-docs/?sQAWUV)

[8. Polson NG, Scott JG. On the Half-Cauchy Prior for a Global Scale Parameter. Bayesian Anal. 2012;7: 887–902. doi:10.1214/12-BA730](https://www.zotero.org/google-docs/?sQAWUV)
