# Supplementary figures and images for: Rapid processing of neutral and angry expressions within ongoing facial stimulus streams: Is it all about isolated facial features?
Source: PLoS One. 2020 Apr 24;15(4):e0231982. doi: 10.1371/journal.pone.0231982 (PMC7182236; doi:10.1371/journal.pone.0231982)

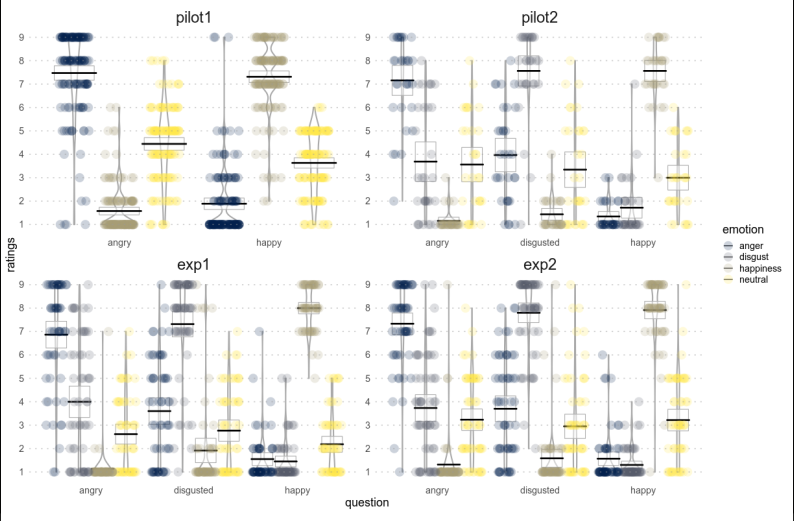

Supplement: S1 Fig — Face ratings for each emotional expression, separately for each participant (single dots), question (how happy/angry/disgusted is this face?), and experiment. Mean values are marked by horizontal black lines and 95% confidence intervals represented as transparent boxes. Ratings range from 1 (very low emotional intensity) to 9 (very high emotional intensity). (TIFF) [file pone.0231982.s002.tiff]

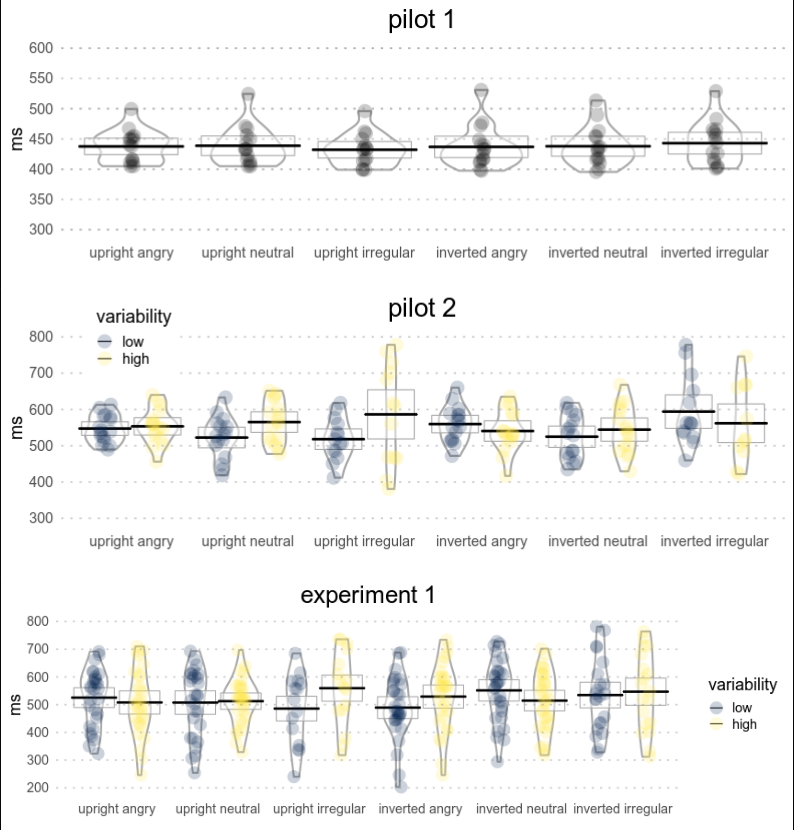

Supplement: S2 Fig — Reaction times (in msec) in response to colored dots during the presentation of each face stream, separately for each participant (single dots), condition, and experiment. Mean values are marked by horizontal black lines and 95% confidence intervals represented as transparent boxes. (TIFF) [file pone.0231982.s003.tiff]

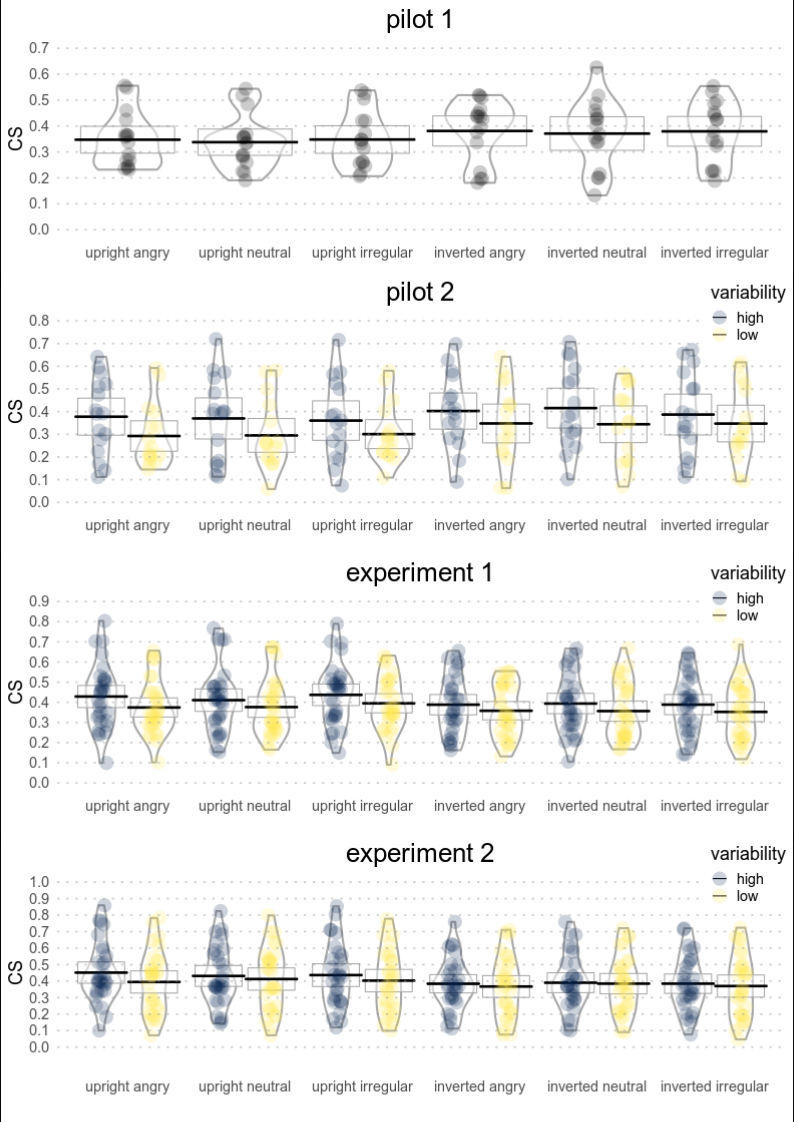

Supplement: S3 Fig — Cosine similarity (CS) calculated at the stimulation frequency for each participant (single dots), condition, and experiment. Mean values are marked by horizontal black lines and 95% confidence intervals represented as transparent boxes. (TIFF) [file pone.0231982.s004.tiff]
